# Information Bottleneck Theory Based Exploration of Cascade Learning

**DOI:** 10.3390/e23101360

**Published:** 2021-10-18

**Authors:** Xin Du, Katayoun Farrahi, Mahesan Niranjan

**Affiliations:** School of Electronics and Computer Science, University of Southampton, Southampton SO17 3AS, UK; K.Farrahi@soton.ac.uk (K.F.); mn@ecs.soton.ac.uk (M.N.)

**Keywords:** information bottleneck theory, Cascade Learning, neural networks

## Abstract

In solving challenging pattern recognition problems, deep neural networks have shown excellent performance by forming powerful mappings between inputs and targets, learning representations (features) and making subsequent predictions. A recent tool to help understand how representations are formed is based on observing the dynamics of learning on an information plane using mutual information, linking the input to the representation (I(X;T)) and the representation to the target (I(T;Y)). In this paper, we use an information theoretical approach to understand how Cascade Learning (CL), a method to train deep neural networks layer-by-layer, learns representations, as CL has shown comparable results while saving computation and memory costs. We observe that performance is not linked to information–compression, which differs from observation on End-to-End (E2E) learning. Additionally, CL can inherit information about targets, and gradually specialise extracted features layer-by-layer. We evaluate this effect by proposing an information transition ratio, I(T;Y)/I(X;T), and show that it can serve as a useful heuristic in setting the depth of a neural network that achieves satisfactory accuracy of classification.

## 1. Introduction

### 1.1. Information Theory and Learning

Pattern recognition is the art and science of finding statistically significant relationships across noisy data, enabling us to associate low level measurements with high level inferences. Measurements, or features derived from measurements, often show variability—systematic and random—in the space they are represented in. Information theory [1,2], characterising the distributions of features and how they relate to the inferences we make, provides tools to understand the performances and quantify the limitations of pattern recognition systems. In classic statistical pattern recognition, information theory has been applied in a number of contexts such as variable splitting in decision trees using mutual information between a variable and a target [3,4]. Early insightful work that is in the same spirit as the topic in this paper include Pearl [5], who use rate distortion theory to explain the performance of pattern recognition systems.

Recent trends in pattern recognition systems that show impressive empirical performance are centred around deep neural network architectures. Though often motivated by our thirst for understanding intelligence by drawing a parallel to neural circuitry in the brain, these approaches derive their power from a rich combination of probabilistic modelling, function approximation, dynamical systems and parallel computing. Recent advances reported in the field of deep neural networks span several challenging problems from visual scene recognition [6] to playing complex games at levels of super-human performance [7].

While many empirical advances in pattern recognition are being reported using deep neural networks, it is widely acknowledged that a theoretical understanding of how this is being achieved is currently lacking. Often, the performances seen reported are counter-intuitive in that very large models of millions of parameters and hundreds of layers are able to generalise to unseen data. Architectural complexity and model capacity, which were seen as leading to over-fitting in the past, appear to be mysteriously helpful in modern applications [8]. Thus, there is much interest in developing tools with which explanations about empirical performances are achieved and how theoretical understandings differ from such results, similar in spirit to Holden and Niranjan [9].

An elegant development in the above direction is the work of Shwartz-Ziv and Tishby [10], who, starting from the information bottleneck theory developed in [11], suggest how we could draw from two mutual information terms to explain learning. Neural networks learn a representation of the inputs in their hidden units and make use of this representation to make predictions about the targets. The fact that mutual information between inputs (*X*) and learned representation (*T*), I(X;T), and that between the representation and subsequent target (*Y*), I(Y;T), evolve during the learning process is at the core of the argument presented in [10]. The primary insight claimed in this work is that, during early stages in learning, networks develop representations that capture information about the targets, maximising the mutual information between learned representations and targets. Beyond a point, networks develop efficient representations by compressing information held in the representation by reducing the mutual information between inputs and learned representations. Referring back to [5], rate distortion theory is about compressing data with minimal loss of information contained in it. The work, done in an era of hardware limitations, focuses on data reduction as an opportunity to save memory needed to store the representation, as opposed to the aim of Shwartz-Ziv and Tishby [10] to see it as an explanation of learning performance.

Though the above is an appealing view of learning dynamics, neatly illustrated using a synthetic dataset, the work attracted many critiques in subsequent literature. Saxe et al. [12] show that the compression observed is dependent on several aspects of the learning setting such as the type of nonlinearity and learning algorithm used. Amjad and Geiger [13] go one step further and are critical of inspiration drawn from the information bottleneck principle to train neural networks. For a more comprehensive summary of the work relating to this topic, we refer the reader to the review article by Geiger [14] and the collection of work published in a recent issue of this journal [15].

### 1.2. Cascade Learning

**Figure 1:**
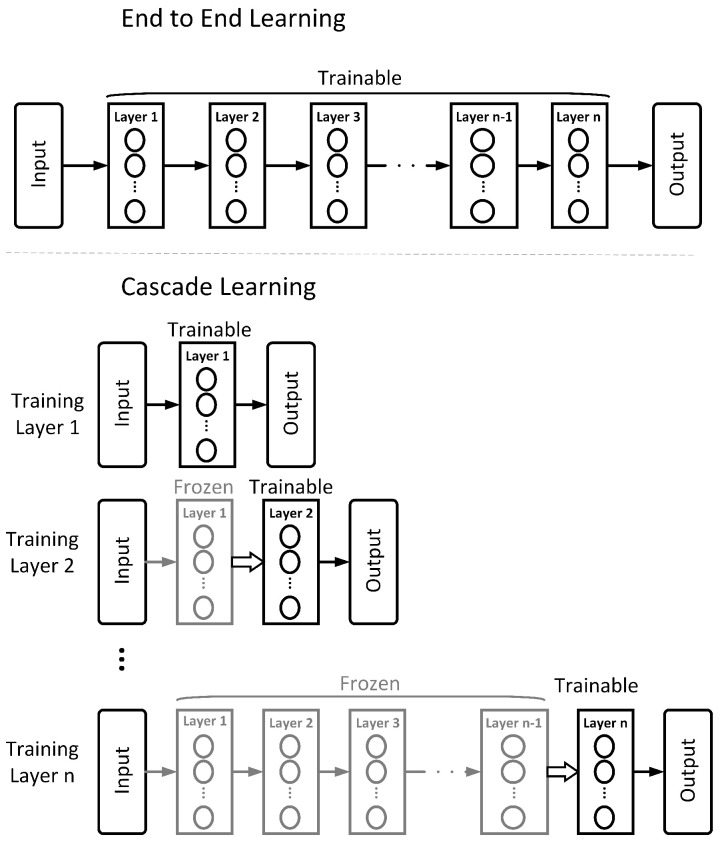
End-to-End (E2E) and Cascade Learning (CL) of multi-layer neural networks. In CL, layers are progressively added and weights of only the most recently added hidden layer and the output layer are trained by gradient descent.

Marquez et al. [16] introduce Cascade Learning (CL), an approach to training neural networks in a layer-wise fashion, inspired by the cascade correlation algorithm of Fahlman and Lebiere [17]. Figure 1 illustrates this approach in comparison to the more classic End-to-End (E2E) method. In CL, a network is trained in a layer-by-layer fashion to gain significant improvements in computation and memory at the expense of marginal accuracy on easy problems (e.g., MNIST and CIFAR 10). Belilovsky et al. [18] illustrate that this layer-wise training can also scale to the much more challenging problem of ImageNet and show comparable performance to popular architectures (e.g., AlexNet and VGG). This is further explored in a comprehensive set of empirical evaluations in Trinh [19]. In our previous work, Du et al. [20], we show that, in addition to computational gains, the nature of packing learned representations into sequentially trained layers gives an advantage in transfer learning problems. Other research on layer-wise training of neural networks include [21,22,23,24]. Raghu et al. [25] show a layer-wise stopping criteria of training an E2E network without loss of performance, which further supports the spirit of layer-wise training.

### 1.3. Contributions

In this work, we side-step the controversial discussions following Shwartz-Ziv and Tishby [10], and ask how information plane trajectories develop during CL. We start by noting that the synthetic classification problem used in [10], being a simple task separable by a single hidden layer network, is unsuitable to make observations about cascade architectures. Hence, we construct four synthetic datasets of architectures similar to [10], but their targets are not separable with a single hidden layer feed-forward network. We also use several benchmark datasets and two widely used problems in character and natural scene recognition. Our novel contributions in this paper are summarised as follows:

1. We show visualisations of how learning dynamics differ between E2E and cascade trained networks on the information plane, illustrating that, by packing information layer-by-layer, we can achieve adequate performance from networks that show no systematic dynamics on the information plane.
2. We note that there is not a direct link between information compression and generalisation, in CL models that achieve the same performance as E2E trained models, thus breaking the overly simplistic link between information bottleneck theory and high empirical performance in deep neural networks.
3. We find that, during CL, the relative changes in the two mutual information terms, the ratio I(Y;T)/I(X;T), make sharp increases when the network develops over-fitting. We propose this as a useful heuristic for automatically controlling the number of layers needed in solving a pattern recognition problem, such adaptive architectures being the original motivation of the cascade correlation algorithm [17].

## 2. Methodology

### 2.1. Datasets

We work with several synthetic and real-world datasets. The construction of the synthetic datasets is inspired by observing that the data used in [10] are an easy problem, learnable by a multi-layer perceptron (MLP) with just a single hidden layer, and hence unsuitable for our purpose of analysing CL. Hence, we generate several MLPs with random weights and an architecture similar to [10] (12-10-7-5-3-1), generated data from them in the 12−dimensional binary input space and train architectures with multiple layers on them (more details see Appendix A). We choose those datasets in which the generalisation performance of a MLP with multiple hidden layers was higher than that of a MLP with a single hidden layer and the imbalance between positive and negative classes was no worse than 33.0%. These are referred to as S17, S28, S44 and S48 in the following discussion. For real-world data, we take four binary classification problems and a multi-class Human activity recognition (HAR) task from the UCI Machine Learning Repository [26], spanning a range in the n,p space where *n* is the number of data items and *p*, the dimensionality of the problem. Some of the datasets have artificially included irrelevant features (having been created for a feature selection competition), and were chosen to observe the robustness of mutual information computations. Additionally, we use MNIST, CIFAR10 and ImageNet, widely used hand-written character and natural image data widely used in the community. Some basic features of the datasets used are shown in Table 1.

### 2.2. Simulation Details

Various network architectures used in our empirical work are given in Table A1 in the Appendix A. Feed-forward networks use hyperbolic tangent activation functions for MLPs and ReLU for Convolutional Neural Networks (CNNs) in their hidden layers. We use a combination of Stochastic Gradient Descent and the Adam optimiser to train networks. We also explore three different methods for estimating mutual information: (a) a discrete binning or histogram approach used by Shwartz-Ziv and Tishby [10]; (b) a pairwise distance (PWD) based approach proposed by Kolchinsky and Tracey [30]; and (c) an Ensemble Dependency Graph Estimator (EDGE) proposed by Noshad et al. [31]. Briefly, the binning approach constructs histograms of the two distributions across which mutual information is measured. The well-known difficulty with this method is the choice of bin/interval size which is a compromise between resolution and data sparsity. Alternate ways of non-parametric density estimation (kernel density estimation [32]) also have an equivalent compromise in setting kernel widths. The Bayesian literature offers a way of combining multiple estimates weighted by priors [33] to cope with bin selection difficulty. Exploring these alternatives is outside the scope of the present work. The method of pairwise distance utilises a Kullback–Leibler divergence based upper bound and a Bhattacharyya distance [34] based lower bound to estimate the mutual information. EDGE is a non-parametric estimator with linear time complexity. To estimate mutual information in this work, we use the binning method for MLPs and EDGE for CNNs. We note that alternative techniques such as Mutual Information Neural Estimation (MINE) [35] and matrix-based estimation [36,37] have also been proposed by other authors, but an exhaustive exploration of the differences was not the objective of this study. With MLPs considered, intermediate layers are of low dimensions (see Table A1), hence binning and PWD methods of estimating mutual information give similar and accurate results. With convolution layers on images, we encounter much larger dimensions. In these, binning and PWD estimation methods find the distributions to be approximately uniform and the estimations become inaccurate. In the calculation of trajectories, we take outputs of each hidden layer (excluding the final classifier) as *T* for estimating two mutual information terms for both CL and E2E networks. For CL, the hidden layer means each trainable layer before the output layer as shown in Figure 1. For E2E, *T* means the outputs of each middle layer.

## 3. Results and Discussion

### 3.1. Information Plane Dynamics of Cascade Learning

**Figure 2:**
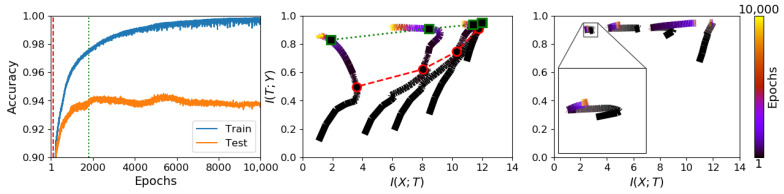
Comparing information plane trajectories for E2E and CL on a synthetic problem (S17). Right panel: the learning curves of an E2E network; middle panel: the information trajectories of an E2E network; and left panel: the information trajectories of a CL network. From left and middle panels, information compression can be observed (between the red and green dashed lines) while the network settles to generalise. Note, however, that such compression can continue into the over-training phase. While E2E learning shows dynamics similar to those in [10], the layer-wise restriction of CL exhibits invisible such dynamics. Also note that, even for E2E training, dynamics observable on simple synthetic problems are not consistently seen in real-world problems. Additional results on other datasets are presented in Appendix A (Figure A2 and Figure A3).

Trajectories on the information plane for CL differ substantially from those of E2E trained models. Figure 2 shows these differences for a synthetic problem. In this we note that E2E training does show the kind of dynamics of rapid learning of the targets, followed by a compression phase, as claimed in [10]. For CL, with weight updates restricted to one layer at a time, the same flexibility does not apply. Hence, while achieving the same performance in solving the task, cascade learned models do not show the same mutual information dynamics.

Furthermore, note that CL trajectories of each layer systematically fall to the left of each other, satisfying the data processing inequality, and the starting points of each layer are only slightly lower than the end point of the previous layer due to their random initialisation and information inheritance from the previous layer. When zoomed in, there is a small amount of information compression to be seen in each layer, but this does not appear significant enough to explain any aspect of learning and generalisation. For additional results on the other synthetic datasets and several real datasets, please see Figure A2 and Figure A3 in Appendix A.

### 3.2. Inconsistency of Information Plane Dynamics on Real Data

We note, looking at the behaviour of real word problems, that the mutual information trajectories rarely show smooth dynamics as in either the simple example constructed in [10], or in any of our synthetic datasets. Figure A3 shows that while, for the fairly simple problem of MNIST, we can observe smooth trajectories, those for the CIFAR10 problem, solved using a convolutional neural network, are very noisy. Apart from the data processing inequality being satisfied layer-wise along the I(X;T) axis, we cannot recognise any consistent behaviour.

### 3.3. Generalisation and Information Compression

We next sought to study information plane dynamics in relation to generalisation. Left and middle panels of Figure 2 show classification performances on training and test sets along with the corresponding information trajectories for the S17 dataset. On these, we can recognise a phase (marked between the red and green vertical lines) during which an increase in test set performance coincides with information compression. However, compression, guided by the training set only, can continue beyond the point at which over-training sets in. The same observation can be made with reference to the other synthetic datasets as shown in Figure A2 in the Appendix A. Such inconsistency casts doubt on the use of information compression as a helpful tool in explaining the high empirical performances seen in deep neural networks.

### 3.4. Information Transition Ratio

**Figure 3:**
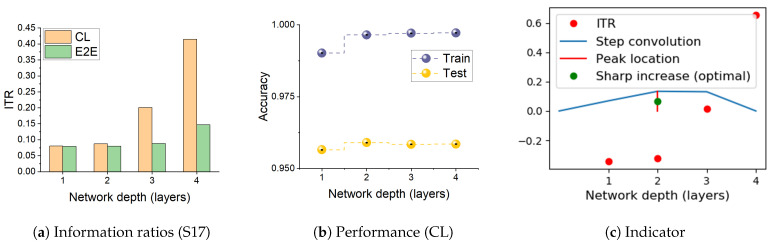

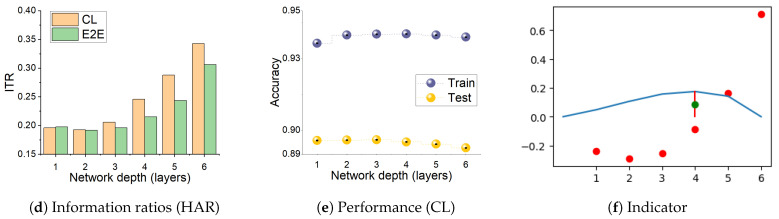
The ratio between information captured about the target, I(T;Y), and representation of the input, I(X;T), computed at different layers for E2E and cascade trained networks. (**a**,**d**) are ratios for S17 and HAR datasets, respectively. (**b**,**e**) show corresponding performance of CL from both training and test sets; (**c**,**f**) provide the indicator of the layer where Information Transition Ratio (ITR) sharp increases or optimal performance of CL can be obtained. For CL, there is often a sharp increase in the ratio, which coincides with over-training (see Performance). Hence, the ratio can be used as a heuristic to determine an optimal depth of a network. Table 2 shows depth of models obtained in this manner for several problems.

**Figure 4:**
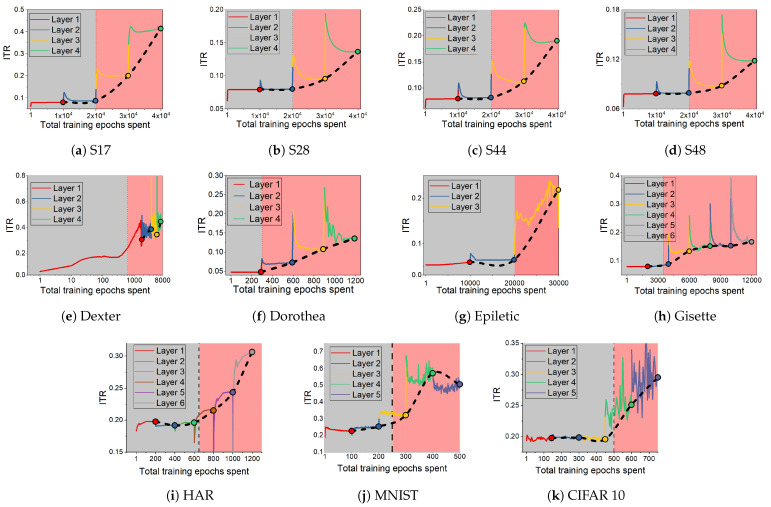
Information ratio I(T;Y)/I(X;T) during CL for synthetic (**a**–**d**) and real datasets (**e**–**k**), shown as a function of total epochs of training. The shading on the figure (gray and pink) shows learning and over-training phases.

We observe above that the dynamics seen on the information plane, compression of I(X;T) in particular, does not necessarily explain generalisation. This appears true not only for E2E training, but also for models with layer-by-layer constrained training that achieve similar performance on the tasks considered. In this section, we note that CL shows an interesting property with respect to the relative speeds with which the two information terms change across the layers. Figure 3 shows the variation in the ratio I(T;Y)/I(X;T), which we refer to as Information Transition Ratio (ITR), computed at different layers for a synthetic (S17) and a real-world (HAR) problem. We compute this ratio for both E2E and cascade trained models and observe that, for CL, a noticeable sharp increase in this ratio coincides with the highest performance. The similar conjunction of accuracy and ITR is obtained on a big computer vision dataset, ImageNet including 23 selected classes, as shown in Figure A1. Figure 4 shows this change for all the problems considered in this paper and, in a majority of them, a sharp increase in ITR is observed. Table 2 shows a summary of this in the various problems considered. The variation in ITR is much smoother for E2E trained models in comparison to CL. This is in line with the nature of the differences between the two approaches. In E2E, there is flexibility in the model to use different parts of the network to distribute different features of the problem, whereas CL hierarchically packs them. Thus, this ratio is unlikely to be useful as a criterion for architecture selection in E2E trained networks.

**Figure 5:**
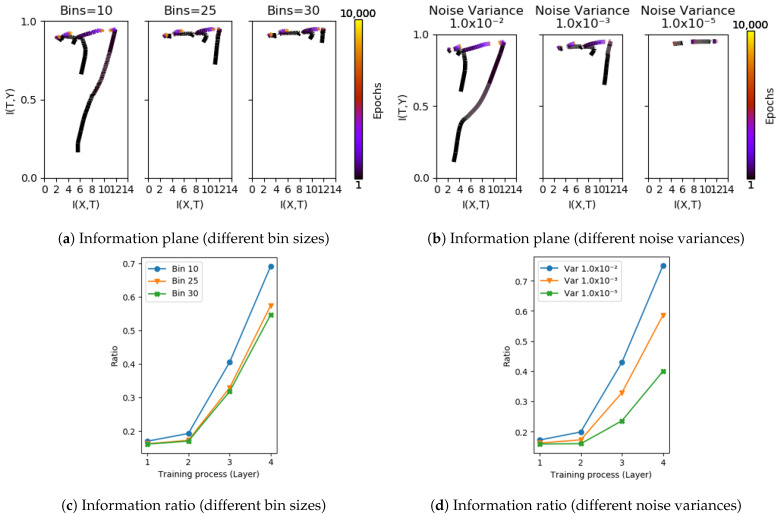
Comparing information plane dynamics and changes in information ratio I(T;Y)/I(X;T) on the S17 dataset. (**a**,**b**) show information planes based on bin and pairwise estimators using different setting of parameters; (**c**,**d**) provide corresponding ITR of layers. Note that, while the trajectories on the information plane are sensitive to the perturbations considered, the rapid increase in the ratio stays fairly stable.

**Figure A1:**
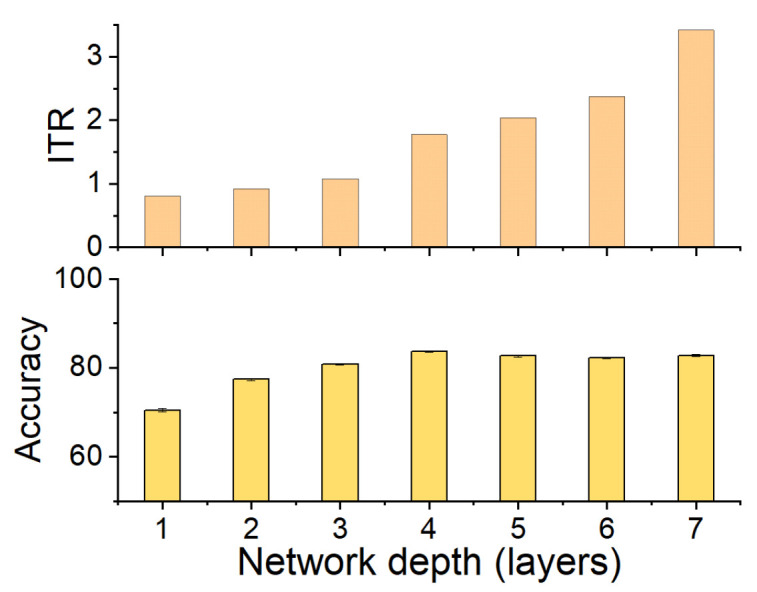
The ITR of CL on the ImageNet dataset. Twenty-three classes are selected in this classification task for saving training time on this large task. The top figure shows a tendency of ITR, and the bottom figure shows performance on test sets, throughout layers of the network. The accuracy achieves an optimal value at the fourth layer where ITR shows a sharp increase. After the fourth layer, the accuracy starts to decrease. The conjunction of testing accuracy decreasing and ITR sharp increasing is consistent with Figure 3.

**Figure A2:**
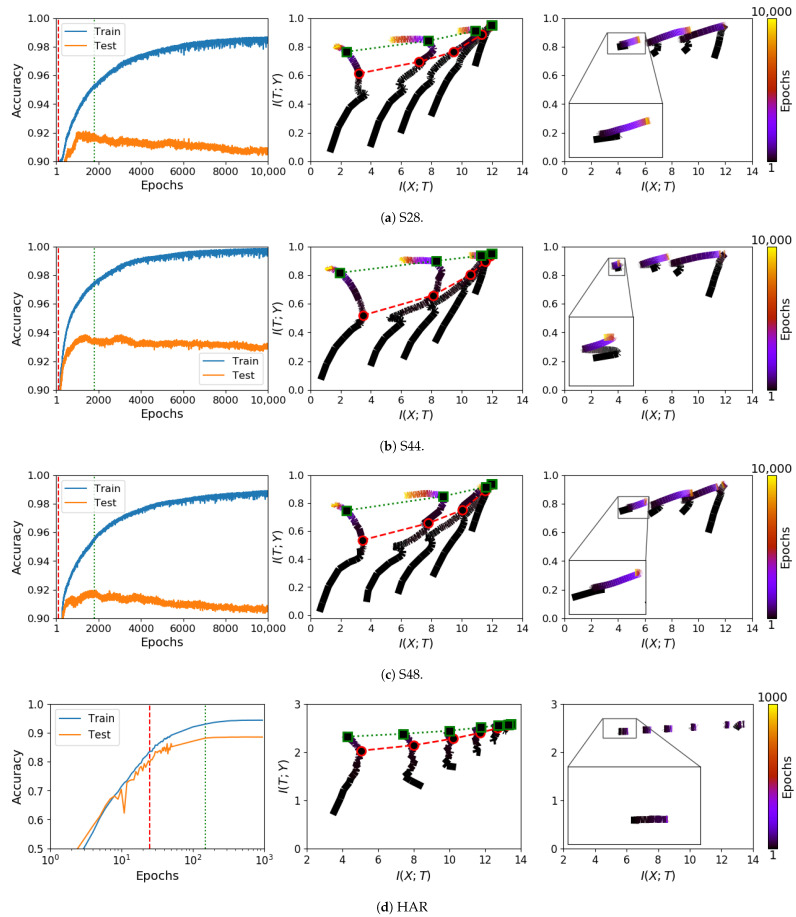
Comparison of information plane dynamics of E2E and CL on three synthetic datasets (**a**–**c**) and (**d**) the HAR dataset with behaviours similar to those discussed in the main text, Figure 2.

**Figure A3:**
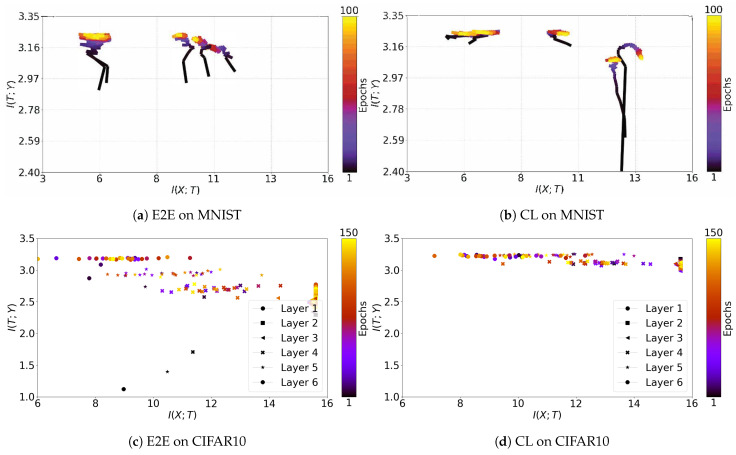
Information plane dynamics on the MNIST and CIFAR10 datasets for E2E and CL. With real data, we rarely see neat and continuous dynamics. The irregular noise-like patterns of the dynamics for the real-world problem CIFAR10 have also been noted in Jónsson et al. [47], where the authors draw the trajectories of each layer in separate graphs.

**Figure A4:**
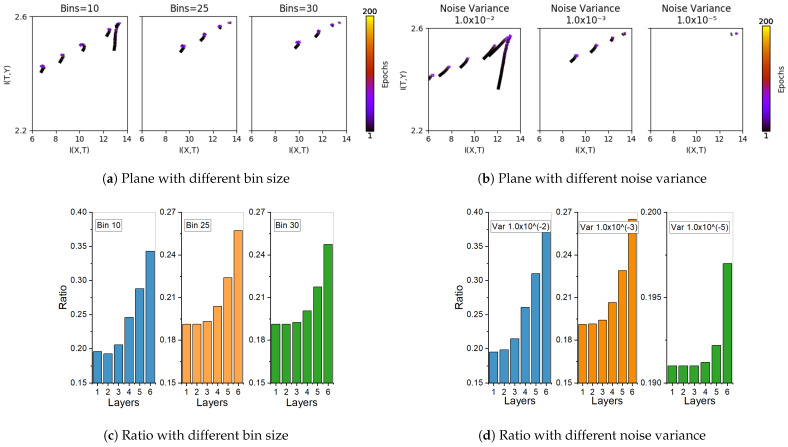
Comparing information plane dynamics and changes in information ratio I(T;Y)/I(X;T) on the human activity recognition (HAR) dataset. Note that, similar to the synthetic data shown in Figure 5, while the trajectories on the information plane are sensitive to the perturbations considered, the rapid increase in the ratio stays fairly stable.

We further observe that the sharp increase in the ratio I(T;Y)/I(X;T) is far more stable than trajectories on the information plane. Figure 5 shows information plane trajectories and the change in ITR taken at different layers of a cascade trained network with (a) different bin sizes in estimating mutual information, and (b) data corrupted by additive noise of different magnitudes. In Figure 5a, we see that the effect of estimating mutual information from different resolutions of bins changes the trajectories significantly. However, the sharp increase in ITR is far more stable across these estimation regimes. We make the same observation when changing the levels of additive noise as seen in Figure 5b,d. The same comparisons on the HAR dataset are shown in Figure A4.

### 3.5. Subspace Visualization

To compare how features evolve across the layers during the two types of training, Figure A6 shows TSNE projections for the HAR problem at a subset of layers and snapshots during learning. As each layer of CL starts from features already learned, early stages of training already show separation between classes in the first few layers, as can be seen by comparing the separation in the first and fourth layers after a single epoch of training. Eventually, in the final layer after 200 epochs of training, the separability in the final layer is nearly identical, making both models achieve the same performance, but along different trajectories of evolution of the way they learn the classes. On the information plane, this maps as the starting point of each layer having relatively high I(Y;T).

### 3.6. Discussion

Shwartz-Ziv and Tishby [10]’s information bottleneck theory approach as a theoretical basis for explaining deep neural network learning has triggered much controversy. These discussions have focused on attributing the causes of the observed compression phase to stochasticity in training [10,38,39], the specific activation function used [12], initialisation of models [40] and the method used for estimating mutual information [41]. Fundamental to much of the discussion in recent literature on this subject is the difficulty of estimating mutual information in high dimensional datasets, which requires accurate models of the probability distributions. The consequence of incorrect estimator selection can lead to incorrect estimation. For example, Schiemer and Ye [41] illustrate information plane dynamics using a six-layer architecture on a synthetic dataset (see Figure 6 of [41]), but the mutual information estimates (I(X;T)) do not satisfy the data processing inequality in the early layers.

#### 3.6.1. Cascade Learning on the Information Plane

The comparison we make between E2E and CL on the information plane is informative. While the former does sometimes develop smooth trajectories showing an increase in I(Y;T) followed by a decrease in I(X;T), this is not consistent on several real-world datasets. Furthermore, in the wide range of examples we considered, it is hard to find convincing evidence of information compression as a plausible explanation for learning and generalisation in deep networks (the original motivation of Shwartz-Ziv and Tishby [10] and others). With synthetic data, specifically constructed to require multiple hidden layers, network layers continue into the information compression phase even after performance on the test data has peaked. On real data, particularly with large-scale problems, it is often difficult to observe smooth trajectories. This could partly be attributed to the difficulties in estimating mutual information, though the various techniques we employed did not result in smooth dynamics.

With CL, we achieve the same level of performance across all the problems considered in this paper. However, with CL, there is very little evidence of information compression or smooth dynamics. This is because training each layer is based on the fixed transformation of data in the previous layer. Therefore, the network has little flexibility to develop the kind of dynamics seen during more flexible E2E training. This suggests that the information plane to observe the relationship across layers has value in explaining how features are learnt, but it is not related to generalisation.

#### 3.6.2. Information Transition Ratio and Network Depth

**Figure A5:**
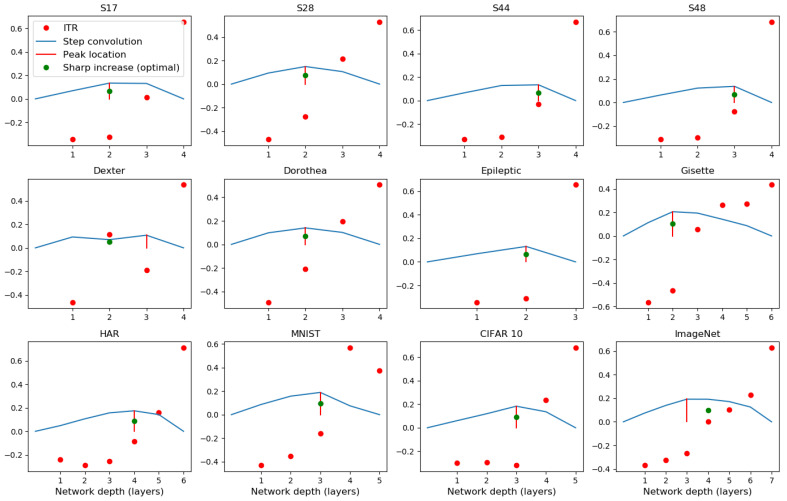
The depth indicator of networks given by the convolution of ITR and a step function on different datasets. Red dots are the values of ITR over layers. Blue solid lines are outputs of convolution of ITR and a step function. Red vertical lines give the location of the peak value of convolution outputs, and the green dots locate the depth of network showing optimal performance or sharp increase of the ITR.

**Figure A6:**
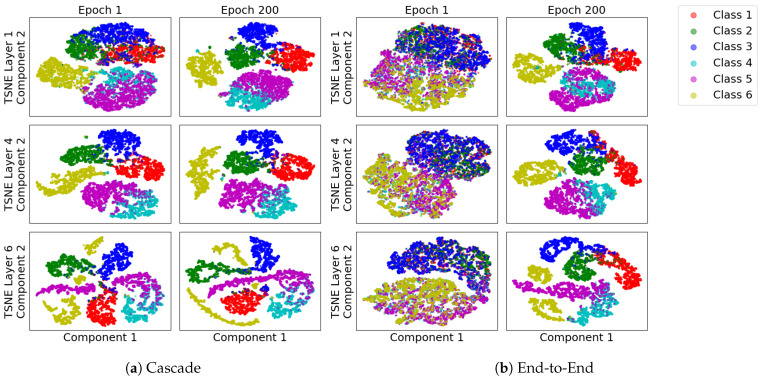
Subspace (TSNE) Visualisation of (**a**) CL and (**b**) E2E on the HAR problem, with layers as rows and three snapshots during training as columns. As each layer of CL starts from features already learned, early stages of training already show separation between classes. On the information plane, this maps as the starting point of each layer having relatively high I(Y;T).

A more important observation we made in this study is that the relative speeds with which the two information terms (I(X;T), the representation term and I(T;Y), the target prediction term) grow between layers can be a useful indicator of generalisation. Increasing the number of layers of a network will reduce the empirical loss monotonically, and is bound to increase I(T;Y). However, training only one layer at a time restricts a network’s flexibility in compression, and hence the reduction in I(T;X) is likely to be relatively smaller. Hence, we see a rapid increase in the ITR when moving into the over-training regime by the addition of more and more layers. We use a step response to find the rapid increase using the convolution of the ITR with a step function. The peak of this convolution output can pinpoint layers at which the ITR will increase sharply (see Figure A5). The calculation of convolution step is simple and can accurately reflect sharp increases in our cases, but this is not the only way to show it. For example, using second derivative of ITR as a function of network depth can also potentially find the sharp change. The sharp increase in ITR as a rule of thumb for selecting depth of a network architecture gives a constructive approach to designing neural networks. We note that, in the majority of the examples considered, the increase coincides with best performance. In a small number of cases where this is not the case, the difference between the automatically selected depth and the best performing depth is just one, and the performance differences between the two is also small. Such a stopping rule has to be seen in the context of the current practice of setting network depths arbitrarily (often in tens to hundreds of layers) followed by E2E training.

#### 3.6.3. Local Objective Function

We note that the work of Wang et al. [42] is perhaps the closest to ours in attempting to understand a layer-wise training setting from the information bottleneck point of view. The authors’ framework of modular training with locally specified objective functions (Noshad et al. [31]) closely parallels CL. They develop an argument as to why early modules (layers) behave in a way so as to lower the overall performance of networks and go on to propose an information theory-based method (referred to as InfoPro) whereby they restrict the growth of I(T;Y) in early layers. Their analyses substantially differ from ours in that, in their work, mutual information is estimated by tagging a linear model (or a generative nonlinear model) between the variables of interest, training the model and using its performance as a proxy for mutual information. This is not a reliable estimator of mutual information, as it is likely to be biased by the choice of such auxiliary models. Furthermore, the argument used in Wang et al. [42] that I(T;Y) is related to generalisation is contradicted by previous authors [43,44]. Duan et al. [45] also address layer-wise learning of neural networks based on kernel machines instead of neurons. Ma et al. [46] propose Hilbert–Schmidt independence criterion (HSIC) bottleneck as an alternative approach to the traditional cross-entropy loss for mitigating exploding and vanishing gradients in training deep neural networks.

## 4. Conclusions

Information theory is an interesting tool to understand the learning process in multi-layer networks. When trained to learn a mapping between input–output data, networks develop complex representations from which predicting the target is easier than from the raw inputs. Shwartz-Ziv and Tishby’s work, illustrated on a simple problem, introduces the idea that representation learning is achieved by information compression during later stages of learning. In this paper, we have taken this framework to help understand information dynamics of a constructive approach by building networks using a layer-wise trained cascade architecture. The dynamics of learning observed on the cascade architecture is, as one would expect, different from that of an E2E trained model, particularly that with the specifics of the original work. Any compression phase we observe is mainly restricted to the early layers, and presumably, once it has extracted useful features, later layers show no such obvious compression for removing the redundancy. We further note that the rate at which the two mutual information terms change has a more robust relationship to generalisation than does the overall dynamics, showing a rapid increase on layers that take the training process into the over-training regime. We propose this as a useful measure to control the depth of a network in CL.

## Figures and Tables

**Table 1:** Datasets used and their summary statistics.

Dataset	(*n*, *p*)	Domain	Input	% Pos	% Neg
S17	(4096,12)	Artificial	Binary	63	37
S28	(4096,12)	Artificial	Binary	62.8	37.2
S44	(4096,12)	Artificial	Binary	37	63
S48	(4096,12)	Artificial	Binary	35	65
Dexter	(600, 20,000)	Text classification	Continuous sparse	50	50
Dorothea	(1150, 100,000)	Drug discovery	Binary sparse	50	50
Epileptic	(11,500, 178)	Epileptic seizure detection	Continuous dense	80	20
Gisette	(6000,5000)	Digit recognition	⋮	30	70
Human activity recognition (HAR) (6-class) [27]	(16,043, 561)	Sensor record	⋮	-	-
MNIST	(70,000, 784)	Image	⋮	-	-
CIFAR-10 [28]	(60,000, 1024)	Image	⋮	-	-
ImageNet [29]	(11,500, 50,176)	Image	⋮	-	-

**Table 2:** Information ratio as a guide in model depth selection.

Dataset	CL		Dataset	CL	
	Ratio ${}^{1}$	Test ${}^{2}$		Ratio ${}^{1}$	Test ${}^{2}$
S17	Layer 3	Layer 2	S28	Layer 3	Layer 2
S44	Layer 3	Layer 2	S48	Layer 3	Layer 2
Dexter	Layer 2	Layer 1/2	Dorothea	Layer 2	Layer 1
Epileptic	Layer 3	Layer 2	Gisette	Layer 3	Layer 2
HAR	Layer 4	Layer 3	MNIST	Layer 3	Layer 2
CIFAR 10	Layer 4	Layer 3/4	ImageNet	Layer 4	Layer 4

${}^{1}$ where the rapid increase of I(T;Y)/I(X:T) (ITR) happens. ${}^{2}$ where the significant performance (over-fitting) occurs on test datasets.

## Data Availability

All synthetic data can be found at [this GitHub page]. The real world data presented in this study are openly available in [UCI Machine Learning Repository] and can be found here [Dexter, Dorothea, Epileptic, Gisette and HAR]. The computer vision data presented in this study are available in [MNIST, CIFAR 10 and ImageNet].

## Appendix A

### Additional Details and Results

As mentioned in Section 2, we use four generated synthetic datasets. For each dataset, a binary set including 4096 12-dimensional data-points is the input of a random initialised network, and the outputs of this network are the targets of the dataset. This process is repeated 50 times using different initialisation for generating 50 datasets using the pytorch platform without training networks. Four of them are selected as synthetic datasets used in this work as they cannot be solved by a single hidden layer network.

We use MLPs and CNNs for different tasks with the optimiser Adam. Different tasks use various initial learning rates and network structures as shown in Table A1. For CIFAR 10 and ImageNet, an auxiliary classifier is used after the convolutional layers. The auxiliary classifier for CIFAR 10 includes two fully connected layers consisting of 64 and 32 units leading to the output layer. For ImageNet, one convolutional layer with 128 units and two fully connected layers with 256 units for each are included. For all of other datasets, the output layer is the classifier and its size is decided by the number of classes of tasks.

**Table A1:** Network architectures and learning rates used for different datasets.

Dataset	Architecture	LR	Dataset	Architecture	LR
Synthetic	[10,7,5,3]	0.001	Epileptic	[32,16,14,12,8,4]	0.01
Dexter	[6,5,4,3]	0.01	Gisette	[32,16,14,10,8,6]	0.001
Dorothea	[20,10,7,3]	0.001	MNIST	[20,20,20,20,20]	0.01
HAR	[10,32,16,10,8,6,4]	0.1	CIFAR 10	layer 1-8:256 layer 9-12:128	0.001
ImageNet	[256,256,256,256,256,256,256]	0.01			

As shown in Figure A5, we use convolve ITR (normalised in the range [0, 1] and mean subtracted) with a step function and use its peak as pointing to a desirable depth of network. Figure A6 shows a visualisation of features learned in each layer for E2E and CL methods of training, using TSNE projection, applied with default parameters in sklearn.manifold library. The visualisation, taken at three snapshots during learning, illustrates the basic difference in the approaches. As opposed to E2E learning, CL forces the layers to be progressively discriminant, whereas E2E distributes the learned information flexibly everywhere. Elsewhere, we see this as a reason that CL trained models are more suitable for transfer learning because they can potentially pack coarse features in early layers. A discussion of this using the human activity recognition problem is in Du et al. [20].
